# Ultrasound elastography: advances and challenges in early detection of breast cancer

**DOI:** 10.3389/fonc.2025.1589142

**Published:** 2025-06-26

**Authors:** Jianmin Zhou, Yanchun Zhang, Shaohua Shi

**Affiliations:** Department of Ultrasound, Yantaishan Hospital, Yantai, Shandong, China

**Keywords:** ultrasound elastography, breast cancer, early detection, artificial intelligence, diagnostic imaging, screening

## Abstract

This review explores recent advances in ultrasound elastography for breast cancer detection, focusing on technological innovations, clinical validation, and emerging challenges in early diagnosis. We analyze how modern elastographic techniques have evolved to address the critical need for accurate, non-invasive breast cancer screening and characterization. Recent methodological developments in ultrasound elastography have significantly enhanced its diagnostic capabilities, particularly in distinguishing malignant from benign breast lesions. We highlight breakthrough technologies including shear wave elastography, strain ratio measurements, and advanced quantitative methods that provide detailed mechanical characterization of breast tissue. The review specifically addresses how these techniques improve the detection of small, early-stage tumors and reduce false-positive rates in dense breast tissue. Artificial intelligence integration has transformed breast elastography workflow, introducing sophisticated pattern recognition and automated lesion characterization. The review also addresses current challenges, including the need for technical standardization, ensuring consistent reproducibility across different settings, managing economic costs, improving accessibility, and developing comprehensive education and training programs for healthcare providers. We analyze emerging solutions, including novel quality assurance protocols and adaptive imaging techniques that accommodate different breast tissue compositions. On summarizing and critically analyzing clinical evidence and technological developments, this review provides a comprehensive perspective on the current state and future directions of breast ultrasound elastography. The integration of advanced elastographic methods with artificial intelligence and standardized protocols promises to establish ultrasound elastography as an essential tool in early breast cancer detection, potentially improving patient outcomes through earlier intervention.

## 1 Introduction

Breast cancer represents one of the most significant global health challenges of the 21st century, with increasing incidence rates that demand more effective and efficient detection methods (1, 2). Current statistics indicate that one in eight women will develop breast cancer during their lifetime (3), emphasizing the critical need for reliable early detection strategies. While mammography continues to serve as the primary screening tool in breast cancer detection, its limitations have become increasingly apparent, particularly in women with dense breast tissue, where sensitivity can decrease significantly (4). In addition, concerns regarding cumulative radiation exposure from regular mammographic screening have prompted the medical community to seek complementary diagnostic approaches that can enhance detection capabilities while minimizing potential risks.

Ultrasound elastography constitutes a non-invasive imaging modality that quantifies tissue stiffness properties, offering supplementary data to conventional B-mode ultrasound. Recent systematic reviews and meta-analyses demonstrate substantial diagnostic capabilities across various breast pathologies. A comprehensive analysis through Hong et al. (5) documented diagnostic precision for non-mass lesions, reporting pooled sensitivity of 79% and specificity of 86% (5). Concurrent evidence from Wang and Wang (6) established analogous accuracy in discriminating triple-negative breast cancer, indicating the technique’s value in characterizing aggressive subtypes (6).

The incorporation of artificial intelligence has also increased ultrasound elastography capabilities. A systematic evaluation through Mao et al. (7) of machine learning applications in elastographic imaging revealed that both traditional computer vision approaches and deep learning models achieve sensitivity ≥80% in tumor classification (7). The development of generative adversarial networks, analyzed by Ansari et al. (8), presents opportunities for generating high-quality elastograms from B-mode ultrasound images, suggesting improved accessibility of this technology (8).

The clinical applications extend beyond primary tumor detection to treatment response monitoring and complication management. Chen et al. (9) demonstrated efficacy in predicting pathological complete response to neoadjuvant chemotherapy (9), while Forte et al. (10) validated its application in breast cancer-related lymphedema diagnosis (10). Additional evidence through Wang et al. (11) substantiated its role in lymph node evaluation, essential for staging and treatment planning (11).

Despite these recent advances, several significant knowledge gaps continue to challenge the successful implementation of ultrasound elastography in clinical practice. A critical concern is the lack of standardization in elastographic techniques and interpretation criteria, as different platforms and institutions often employ varying methodologies, making result comparison and protocol establishment difficult. The integration of multi-parametric approaches, which aim to combine various elastographic methods with other imaging modalities, requires further research to maximize diagnostic accuracy and clinical utility. The optimization of artificial intelligence algorithms for real-time clinical application remains an ongoing challenge. While these computational tools show considerable promise, further refinement is necessary to ensure they can deliver reliable, instantaneous results during patient examinations. The field also faces challenges in validating elastographic markers for early detection in high-risk populations, a key step in establishing this technology’s role in preventive medicine. Comprehensive cost-effectiveness studies are essential to demonstrate the economic viability and healthcare benefits of implementing these techniques in screening programs, particularly as healthcare systems worldwide seek evidence-based justification for adopting new technologies.

This review aims to comprehensively evaluate the current state of ultrasound elastography in breast cancer detection, with particular emphasis on its clinical applications, implementation challenges, and future directions. We systematically analyze the evolving role of different elastographic techniques in breast imaging, examining their diagnostic performance, technical considerations, and integration into existing clinical workflows. Special attention is devoted to addressing critical knowledge gaps, including the standardization of acquisition protocols and interpretation criteria across different ultrasound platforms, the potential of multi-parametric imaging approaches, and the emerging role of artificial intelligence in real-time elastographic analysis. On critically assessing current limitations and technical challenges, this review provides insights into practical implementation strategies while identifying areas requiring further research to enhance the clinical utility of elastography in breast cancer screening and diagnosis. Furthermore, we evaluate the economic implications and cost-effectiveness considerations that influence the widespread adoption of elastographic techniques in breast imaging protocols, aiming to provide evidence-based recommendations for clinical practice optimization.

## 2 Technical principles and methods in ultrasound elastography

### 2.1 Basic principles

Ultrasound elastography encompasses two fundamental approaches: Strain Elastography (SE) and Shear Wave Elastography (SWE), each offering distinct advantages and applications in breast cancer detection (12). SE, the earlier of the two techniques, operates on the principle of tissue deformation in response to external or physiological forces. This method evaluates the relative displacement of tissue elements before and after compression, generating qualitative or semi-quantitative assessments of tissue stiffness (13). The technique relies on the principle that softer tissues deform more readily than stiffer tissues under the same applied force.

SWE, representing a more recent technological advancement, uses acoustic radiation impulse to generate shear waves within the tissue (14). By tracking the propagation speed of these waves, SWE enables direct quantitative measurement of tissue stiffness, expressed in kilopascals (kPa) or meters per second (m/s). This quantitative capability represents a significant advantage over strain elastography, providing objective measurements that facilitate standardization and comparison across different examinations and institutions.

The physical principles underlying these techniques are rooted in fundamental mechanical concepts (15, 16). In SE, the stress-strain relationship of tissues follows Hooke’s Law within the elastic limit, though biological tissues often exhibit more complex non-linear behaviors. SWE exploits the relationship between shear wave propagation speed and tissue stiffness, described by the equation E = 3ρc², where E represents Young’s modulus, ρ is tissue density, and c is the shear wave speed.

### 2.2 Measurement techniques

The implementation of elastographic measurements involves sophisticated hardware and software solutions that have evolved significantly over time. Modern systems incorporate advanced processing algorithms to optimize image quality and measurement accuracy. In strain elastography, real-time feedback mechanisms help operators maintain consistent compression techniques, while SWE systems employ complex beam forming sequences to generate and track shear waves effectively.

The measurement parameters in elastography have been refined through extensive clinical research and validation studies (17–19). Maximum stiffness (Emax) has emerged as a particularly valuable metric in distinguishing malignant from benign lesions, with established threshold values that vary somewhat among different system manufacturers but generally fall within the range of 80–120 kPa for malignancy. Mean stiffness (Emean) provides complementary information about overall lesion characteristics, while the stiffness ratio between lesion and surrounding tissue helps account for individual variations in background tissue properties.

Pattern recognition has become increasingly important in elastographic assessment, with certain features showing high specificity for malignancy. The “stiff rim” sign, characterized by a ring of increased stiffness surrounding a lesion, has been particularly well-documented as an indicator of malignant potential (20). These qualitative features, when combined with quantitative measurements, enhance diagnostic accuracy and help guide clinical decision-making.

## 3 Clinical applications of ultrasound elastography in breast cancer

### 3.1 Diagnostic performance

The diagnostic capabilities of ultrasound elastography extend far beyond basic sensitivity and specificity metrics. Recent multicenter trials have revealed nuanced performance characteristics across different patient populations and clinical scenarios (21–23). Age-stratified analyses have revealed interesting patterns in elastographic performance. In women under 40, where dense breast tissue often complicates traditional imaging, elastography shows enhanced performance with a negative predictive value of 98.2% for BI-RADS 3 lesions (24). BI-RADS (Breast Imaging Reporting and Data System) is a standardized system used to classify findings in breast imaging exams like mammography and ultrasound, estimating the risk of breast cancer. This high negative predictive value has significant implications for reducing unnecessary biopsies in younger women, where the psychological impact and cost considerations of false positives are particularly relevant.

The integration of elastographic criteria into the BI-RADS classification system has led to refined risk stratification models. Studies show that incorporating elastographic features can reclassify up to 40% of BI-RADS 4a lesions as BI-RADS 3 (19), potentially reducing biopsy rates without missing clinically significant cancers. This reclassification has been validated through long-term follow-up studies showing less than 0.2% false-negative rates in downgraded lesions.

### 3.2 Lesion characterization

Advanced elastographic analysis has revealed distinct mechanical signatures for various breast pathologies. Recent studies using artificial intelligence-enhanced pattern recognition have identified specific elastographic “fingerprints” for different histological subtypes:

Ductal Carcinoma in Situ (DCIS) presents unique challenges in elastographic assessment, but recent advances in high-resolution elastography have improved detection capabilities (25, 26). DCIS typically shows a distinctive “honeycomb” pattern on strain elastography, with mean stiffness values ranging from 80–120 kPa on shear wave imaging. The presence of microcalcifications within DCIS creates characteristic mechanical heterogeneity that can be quantified through advanced texture analysis.

Triple-negative breast cancers demonstrate particularly high stiffness values, often exceeding 180 kPa, with distinct peripheral stiffness patterns that correlate with their aggressive biological behavior (27, 28). These tumors often show a “stiff rim” sign, which has been associated with increased likelihood of lymphovascular invasion and poorer prognosis.

HER2-positive tumors exhibit unique elastographic features, including increased internal heterogeneity and specific strain patterns that correlate with tumor grade. Studies have shown that the ratio of peripheral to central stiffness in HER2-positive lesions can predict response to targeted therapies with high accuracy (29, 30).

Rare histological subtypes such as metaplastic carcinoma and adenoid cystic carcinoma show characteristic elastographic patterns that aid in preoperative diagnosis. Metaplastic carcinomas typically display extreme stiffness values (>200 kPa) with internal areas of softening corresponding to matrix production, while adenoid cystic carcinomas show moderate stiffness with distinctive internal architectures (31, 32).

### 3.3 Treatment monitoring

Ultrasound elastography has emerged as a valuable tool for monitoring breast cancer treatment response, offering real-time assessment of tumor mechanical properties throughout the therapeutic journey. This capability provides clinicians with crucial information about treatment efficacy and helps guide therapeutic decisions, particularly in the context of early detection and intervention strategies.

During the initial phases of treatment, elastography enables early detection of therapeutic response by measuring changes in tissue stiffness before conventional imaging shows morphological changes. Studies have demonstrated that successful chemotherapy typically results in decreased tissue stiffness, detectable by elastography as early as 1–2 weeks into treatment (33, 34). This early assessment capability allows clinicians to identify non-responding patients quickly, make timely adjustments to treatment protocols, and reduce unnecessary exposure to ineffective treatments, ultimately optimizing patient outcomes through personalized therapeutic approaches.

Modern elastographic techniques provide several quantitative parameters for treatment monitoring, including shear wave velocity measurements, strain ratio comparisons, elastic modulus values, and tissue deformation patterns. These parameters offer objective metrics for tracking treatment progress, with research indicating that a reduction in shear wave velocity values exceeding 30% often signifies positive treatment response. Furthermore, changes in strain ratios have shown strong correlation with pathological response, while decreases in elastic modulus typically precede observable reductions in tumor size, providing earlier indicators of treatment effectiveness.

Elastography has demonstrated particular value in monitoring neoadjuvant chemotherapy response, where early assessment of treatment efficacy is essential for surgical planning (35). The ability to quantitatively measure changes in tissue stiffness throughout the course of therapy enables clinicians to make informed decisions about the timing and extent of surgical intervention. This capability has proven especially valuable in cases where traditional imaging modalities may provide ambiguous results, offering an additional layer of confidence in treatment response assessment and surgical decision-making.

The integration of elastographic monitoring with conventional imaging techniques has created a more comprehensive approach to treatment assessment, enabling clinicians to correlate mechanical property changes with morphological and functional alterations in breast tissue. This multimodal approach enhances the accuracy of response evaluation and provides a more complete picture of disease progression or regression, ultimately contributing to more effective and personalized treatment strategies for breast cancer patients (36, 37).

**Figure 1:**
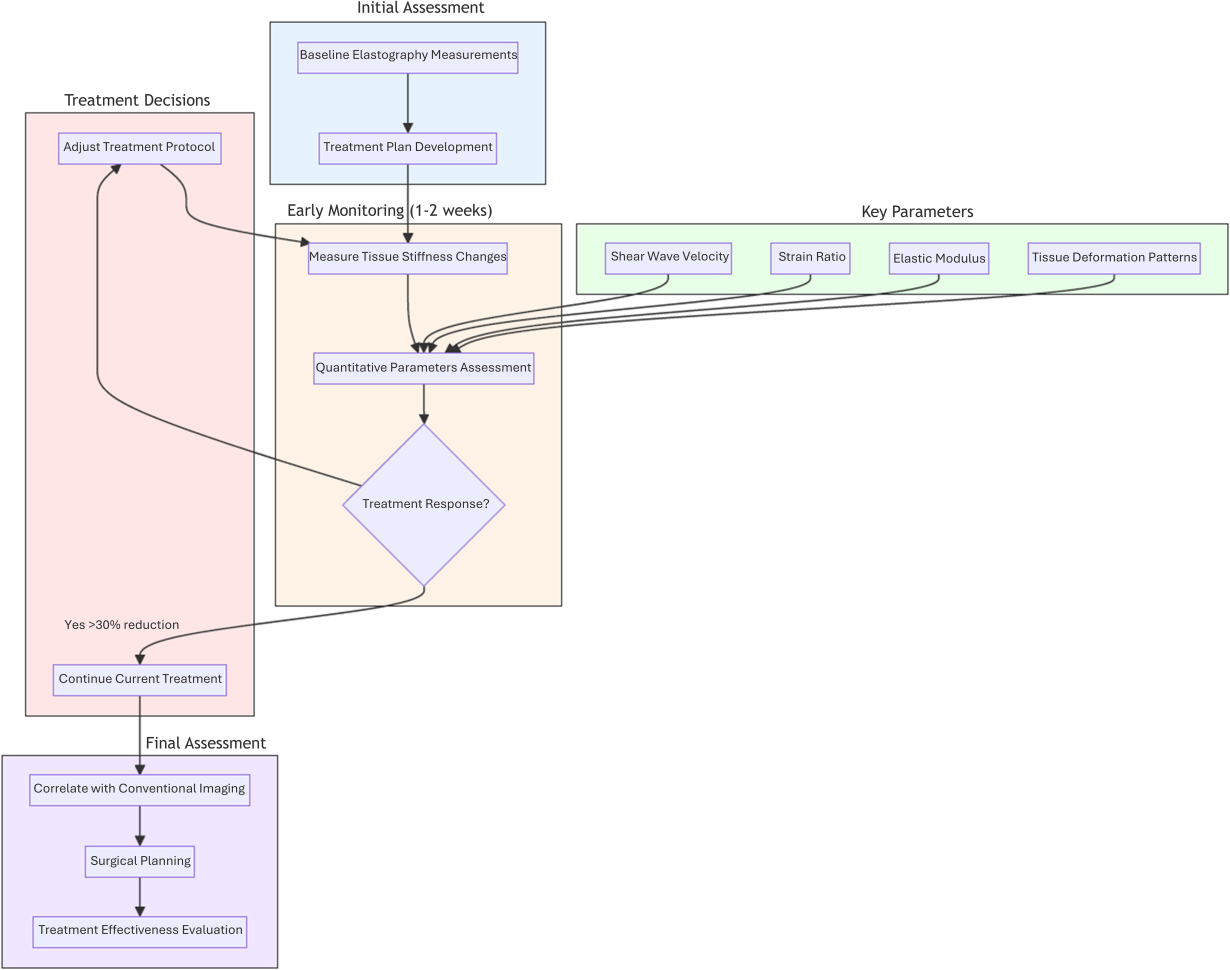
Schematic representation of ultrasound elastography-based treatment monitoring workflow in breast cancer management. This figure illustrates a systematic approach to evaluating treatment response through ultrasound elastography, emphasizing key phases and decision points: Initial Assessment (blue), Early Monitoring Phase (orange), Treatment Decisions (red), Final Assessment Phase (purple).

**Figure 1** illustrates the systematic approach to monitoring breast cancer treatment response using ultrasound elastography. The process begins with initial assessment (blue), establishing baseline elastographic measurements essential for subsequent comparative analysis. During the early monitoring phase (orange), typically within 1–2 weeks of treatment initiation, changes in tissue stiffness are quantitatively evaluated through multiple parameters (green), including shear wave velocity, strain ratio, elastic modulus, and tissue deformation patterns. A key decision point (red) occurs based on treatment response, where a reduction in shear wave velocity exceeding 30% indicates positive response, prompting continuation of the current treatment protocol. Insufficient response triggers treatment protocol adjustments, leading to renewed monitoring. The final assessment phase (purple) integrates elastographic findings with conventional imaging modalities, facilitating informed surgical planning and comprehensive treatment effectiveness evaluation. This systematic approach enables real-time assessment of therapeutic efficacy and supports personalized treatment adjustments, ultimately optimizing patient outcomes through evidence-based decision-making.

## 4 Advanced techniques and developments in ultrasound elastography for breast cancer

### 4.1 Novel elastographic methods

The landscape of breast cancer detection has been transformed by revolutionary advances in ultrasound elastography, where emerging technologies seamlessly blend to create increasingly sophisticated diagnostic capabilities. At the forefront of these innovations, multi-frequency shear wave elastography has redefined the boundaries of spatial resolution, enabling the visualization of microscopic tissue changes and detection of lesions as small as 3mm (38, 39). This breakthrough naturally paved the way for the development of three-dimensional elastography mapping, which extends these capabilities across the entire breast volume, providing a comprehensive view of tissue mechanical properties that prove particularly valuable in dense breast tissue evaluation (23).

The evolution of these technologies has been dramatically accelerated by the integration of artificial intelligence, giving rise to AI-driven elastography (40). This sophisticated fusion of machine learning algorithms with real-time elastographic data has transcended traditional limitations, not only enhancing diagnostic accuracy but also reducing operator dependency (41). Building upon this foundation, dual-mode elastography emerged as a natural progression, ingeniously combining strain and shear wave techniques to capture both quasi-static and dynamic mechanical properties simultaneously. This synergistic approach provides a more nuanced understanding of tissue characteristics, particularly valuable in complex cases where traditional methods might yield ambiguous results.

The pursuit of even greater precision led to the development of micro-elastography, pushing the boundaries of resolution to the cellular level through high-frequency ultrasound exceeding 20 MHz (42, 43). This microscopic insight into tissue mechanical properties represents a quantum leap in early detection capabilities, allowing clinicians to identify malignant transformations before they manifest as visible morphological changes. Complementing this advancement, contrast-enhanced elastography emerged as a sophisticated hybrid technique, integrating mechanical property assessment with vascular imaging to provide a comprehensive evaluation of tissue characteristics (44, 45).

These interconnected technological innovations form a cohesive framework that has fundamentally transformed the approach to breast cancer detection. Each advancement builds upon and enhances the others, creating a synergistic system that offers unprecedented accuracy in early detection and characterization of breast lesions. As these technologies continue to evolve and converge, they are reshaping clinical practices and setting new standards in breast cancer screening and diagnosis. The remarkable fusion of these complementary approaches not only improves diagnostic accuracy but also provides clinicians with a more complete understanding of tissue pathology, ultimately leading to earlier detection and better patient outcomes. This technological renaissance in elastography represents not just a series of individual improvements, but a fundamental shift in how we approach breast cancer detection, promising a future where early diagnosis becomes increasingly precise and accessible.

**Table 1** summarizes the advanced techniques and developments in ultrasound elastography for breast cancer detection.

**Table 1:** Cutting-edge developments in elastography, highlighting their key features, benefits, and technical aspects.

Advanced Technique	Key Features	Benefits	Technical Specifications	References
Multi-frequency Shear Wave Elastography	Enhanced spatial resolution Microscopic tissue change detection	Detection of lesions as small as 3mm Improved visualization	High-frequency ultrasound capabilities	(38, 39)
3D Elastography Mapping	Volumetric tissue assessment Comprehensive breast coverage	Effective in dense breast tissue Complete tissue mechanical property view	Full-volume scanning capabilities	(23)
AI-driven Elastography	Machine learning integration Real-time data processing	Enhanced diagnostic accuracy Reduced operator dependency	Advanced algorithmic processing	(40, 41)
Micro-elastography	Cellular-level resolution High-frequency ultrasound (>20 MHz)	Early malignant transformation detection Pre-morphological change identification	>20 MHz frequency ultrasound	(42, 43)
Contrast-enhanced Elastography	Integrated mechanical and vascular imaging Hybrid assessment technique	Comprehensive tissue evaluation Enhanced characterization capabilities	Combined contrast and elastography systems	(44, 45)

Each technique represents a significant advancement in breast cancer detection technology, contributing to improved diagnostic capabilities and patient outcomes.

### 4.2 Artificial intelligence integration

The integration of artificial intelligence with ultrasound elastography has revolutionized early breast cancer detection, creating a sophisticated diagnostic paradigm that enhances both the acquisition and interpretation of elastographic data (46). Deep learning algorithms have transformed conventional elastographic imaging into an intelligent diagnostic tool that can detect subtle tissue changes indicative of early malignancy, marking a significant advancement in breast cancer screening capabilities (47).

The foundation of this technological synergy lies in specialized convolutional neural networks designed to analyze elastographic patterns specific to breast tissue (48). These advanced systems process complex layers of mechanical property data, detecting minute variations in tissue stiffness that often precede visible anatomical changes (49). On learning from vast databases of confirmed cases, these AI systems have developed a remarkable ability to distinguish the mechanical signatures of early-stage malignancies from benign tissue alterations, significantly improving the accuracy of early detection.

Real-time AI processing has fundamentally transformed the breast examination workflow, providing immediate feedback during ultrasound elastography procedures. The system continuously analyzes incoming data streams, guiding sonographers to achieve optimal imaging parameters and ensuring consistent pressure application (50). This interactive guidance has particularly enhanced the detection of small, early-stage lesions that might be overlooked in conventional examinations, while simultaneously reducing operator dependency and improving the reproducibility of results (51).

The AI framework’s ability to standardize elastographic measurements across different operators and equipment has addressed a major challenge in early breast cancer detection. By automatically compensating for variations in technique and patient characteristics, these systems ensure consistent and reliable results across multiple examinations (52). This standardization has proven especially valuable in monitoring subtle changes over time, enabling the detection of developing malignancies through comparative analysis of sequential examinations.

A particularly significant advancement lies in the AI system’s capability to perform multi-parametric analysis, integrating elastographic data with conventional ultrasound features and clinical information (53). This comprehensive approach combines mechanical properties, morphological characteristics, and vascular patterns to create detailed tissue characterization profiles. The sophisticated algorithms weigh these multiple parameters simultaneously, significantly improving the accuracy of early-stage cancer detection and reducing false-positive rates in screening programs.

The evolution of AI in breast elastography continues to push the boundaries of early detection capabilities. Emerging developments in deep learning architectures and neural network design promise even more sophisticated analysis of tissue mechanical properties. These advancements suggest a future where AI-enhanced elastography becomes an increasingly precise tool for early breast cancer detection, capable of identifying malignancies at their earliest and most treatable stages. The ongoing refinement of these technologies, coupled with their growing integration into clinical practice, represents a significant step forward in the fight against breast cancer, offering new hope for earlier detection and improved patient outcomes.

**Figure 2:**
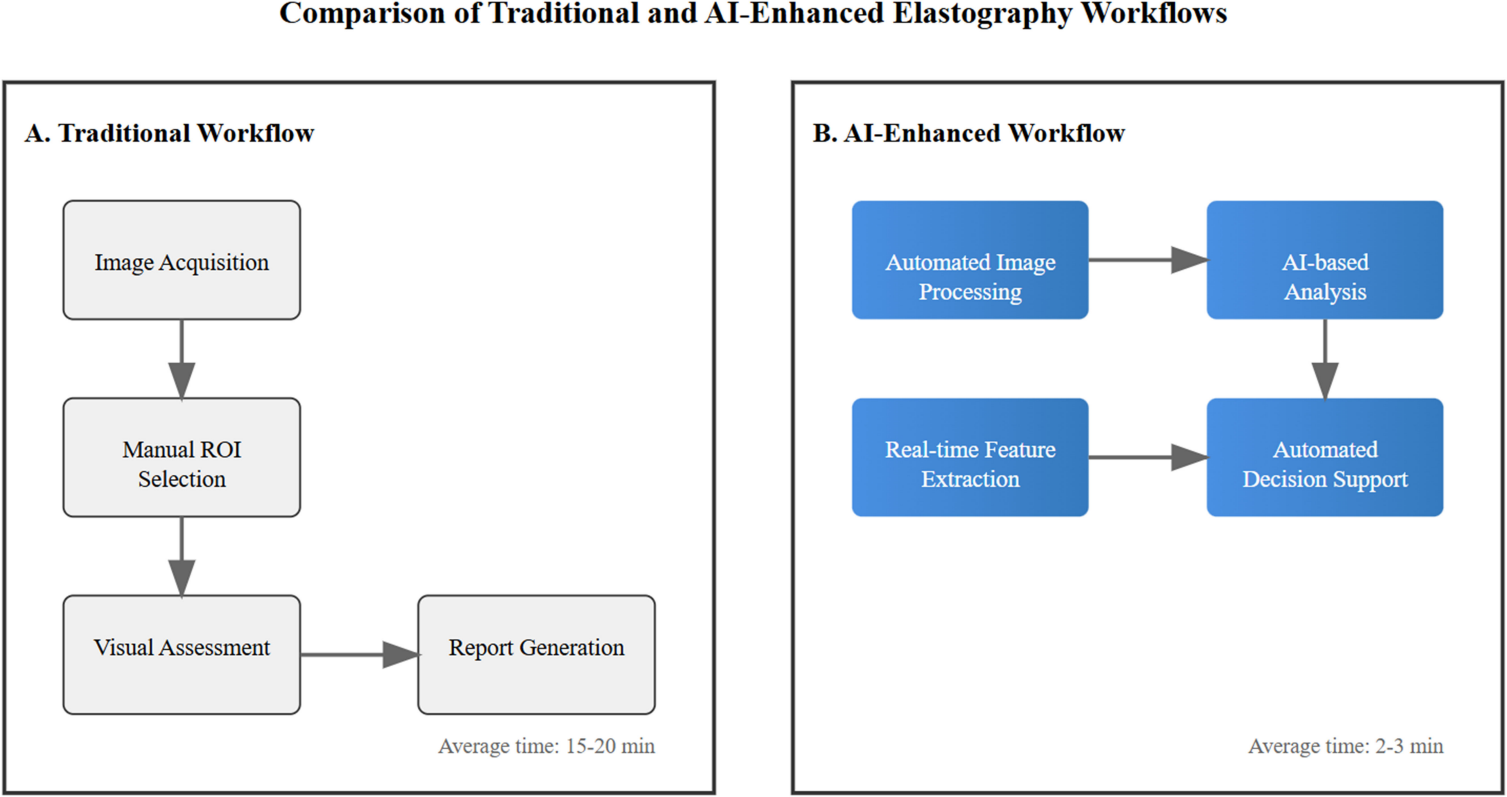
Comparison between traditional manual workflow **(A)** and AI-enhanced workflow **(B)** in breast elastography analysis. Blue gradient boxes indicate AI-automated processes. Arrows show workflow progression.

**Figure 2** illustrates the transformative impact of artificial intelligence integration in breast elastography workflow. This comparative visualization demonstrates the evolution from traditional manual processes to AI-enhanced automation, highlighting key improvements in efficiency and standardization. The AI-enhanced workflow represents a significant advancement in breast elastography analysis through multiple key improvements. The most notable impact is the dramatic reduction in examination time, decreasing from 15–20 minutes to just 2–3 minutes, representing approximately an 85% improvement in temporal efficiency. This automation fundamentally addresses the challenge of operator variability by implementing standardized analysis protocols, ensuring consistent and reproducible results across different clinical settings and operators. The integration of real-time decision support systems further enhances the diagnostic process, providing immediate, data-driven insights to support clinical judgment. Moreover, the streamlined workflow significantly improves overall clinical efficiency, allowing for higher patient throughput without compromising diagnostic quality. This transformation from traditional to AI-enhanced methodology not only optimizes resource utilization but also potentially improves diagnostic accuracy through standardized quantitative analysis, ultimately contributing to better patient care and clinical outcomes.

### 4.3 Multi-modality integration

The integration of ultrasound elastography with complementary imaging modalities has created a powerful approach to early breast cancer detection, transcending the limitations of individual techniques. This sophisticated fusion of imaging technologies has established a comprehensive diagnostic framework that combines the mechanical property assessment capabilities of elastography with the anatomical detail of conventional imaging methods, significantly enhancing the accuracy and reliability of early breast cancer detection (54).

At the forefront of this multi-modal approach is the integration of shear wave elastography with high-resolution B-mode ultrasound, creating a unified imaging platform that simultaneously captures tissue stiffness and morphological characteristics (55). This combination provides clinicians with real-time, co-registered information about tissue mechanical properties and structural features, enabling more precise characterization of suspicious lesions at their earliest stages (56). The correlation between elastographic patterns and conventional ultrasound features has proven particularly valuable in differentiating early malignant changes from benign alterations, significantly improving diagnostic confidence and reducing unnecessary biopsies.

The incorporation of contrast-enhanced ultrasound into the elastographic assessment has further enriched the diagnostic capabilities of this multi-modal approach (57). By simultaneously evaluating tissue stiffness, morphology, and vascular patterns, clinicians can now access multiple aspects of tissue pathology in a single examination. This comprehensive evaluation has proven especially valuable in detecting small, early-stage tumors that might be missed by individual imaging modalities alone, as the combination of mechanical and vascular information provides a more complete picture of tissue health (58).

Advanced image fusion techniques have enabled the precise co-registration of elastographic data with mammographic and magnetic resonance images, creating a powerful multi-parametric assessment tool (59). This integration allows clinicians to correlate areas of increased stiffness with suspicious findings on other imaging modalities, providing a more complete understanding of tissue characteristics and spatial relationships (60). The ability to simultaneously view and analyze complementary imaging data has significantly enhanced the detection of subtle tissue changes that might indicate early malignant transformation.

The temporal dimension of multi-modal imaging has introduced new possibilities in breast cancer surveillance. On tracking changes across multiple imaging parameters over time, clinicians can now detect subtle variations that might indicate developing pathology (61). This longitudinal approach, combining elastographic measurements with other imaging biomarkers, has proven particularly valuable in monitoring high-risk patients and evaluating response to preventive interventions, enabling more timely and targeted clinical decisions.

**Figure 3:**
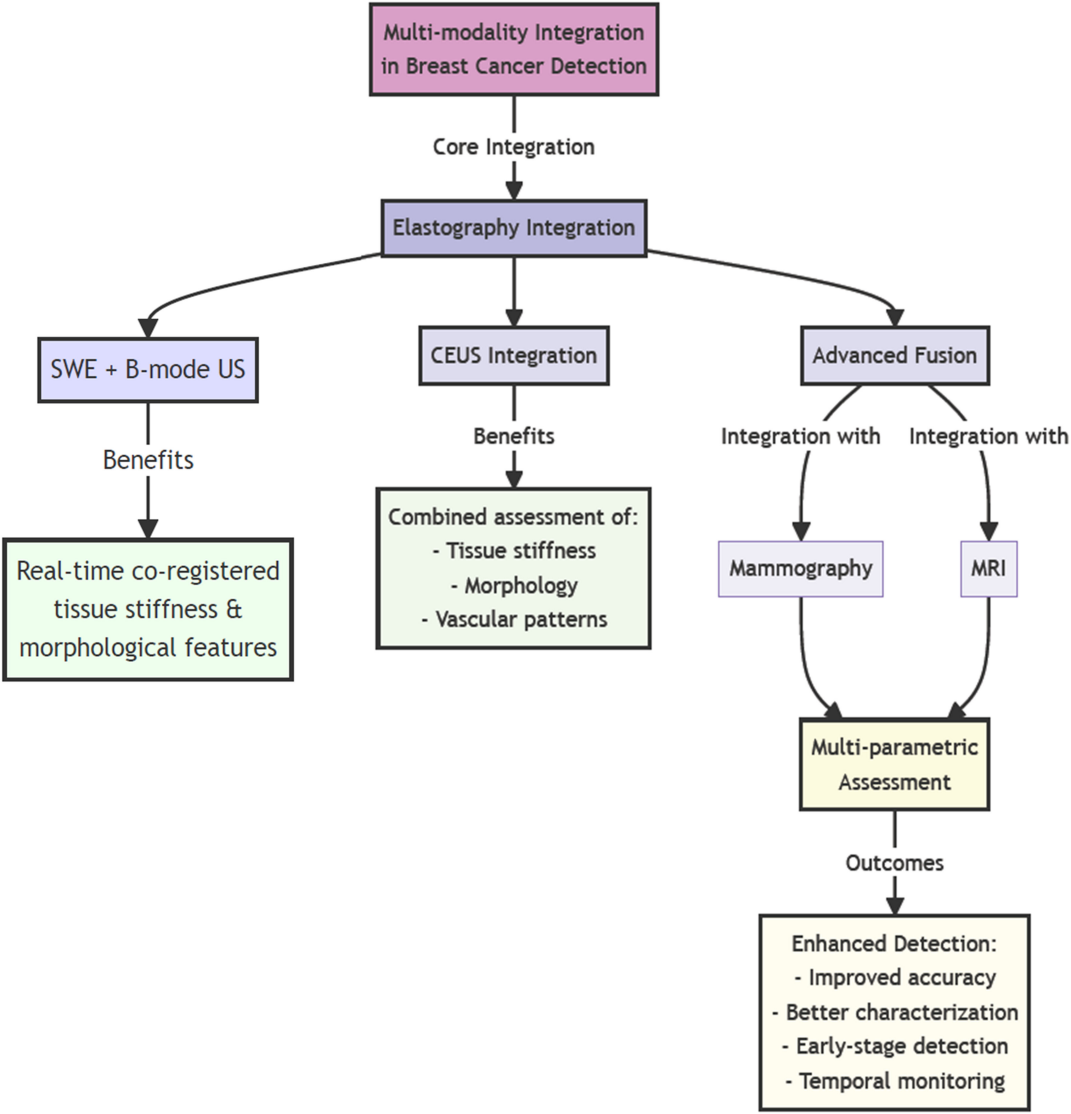
Schematic representation of the multi-modal imaging integration framework for breast cancer detection. (high resolution image is provided in Supplementary Material).

**Figure 3** provides a comprehensive overview of the multi-modal integration framework discussed in Section 4.3. The hierarchical structure demonstrates how various imaging technologies converge to create a more robust diagnostic approach. As illustrated in the flowchart, the integration of elastography with conventional imaging modalities creates multiple pathways for improved diagnostic accuracy. The diagram particularly emphasizes the synergistic relationships between different imaging techniques, showing how their combination addresses the limitations of individual modalities. This integrated approach, as depicted in the figure, enables clinicians to simultaneously evaluate multiple tissue characteristics, leading to more accurate and reliable early breast cancer detection.

## 5 Future directions and challenges

The evolution of ultrasound elastography in breast cancer detection faces both promising opportunities and significant challenges that will shape its future implementation. As we look ahead, several critical areas require attention and development to fully realize the potential of this technology in early detection of breast cancer.

### 5.1 Technical standardization and reproducibility

Standardization and reproducibility of elastographic measurements are significant challenges that affect the clinical utility and adoption of breast elastography. These issues arise from interconnected factors that influence the reliability and consistency of diagnostic outcomes, necessitating systematic attention and innovative solutions.

A key challenge is the operator dependency inherent in elastographic techniques. The application of appropriate compression force, accurate probe positioning, and consistent scanning techniques heavily relies on the operator’s expertise. Even experienced sonographers can produce markedly different results due to subtle variations in technique, particularly in strain elastography, where manual compression affects image quality and measurement accuracy (62).

Patient-related factors further complicate standardization by introducing biological variability. Differences in breast tissue composition, tissue depth, and anatomical variations among patients significantly impact elastographic measurements. Physiological factors such as hormonal status, age-related changes, and prior surgical interventions also influence tissue elasticity, complicating the establishment of universal reference values and standardized interpretation criteria.

To address these challenges, comprehensive standardization protocols have been developed, covering all aspects of elastographic examination. These protocols focus on optimizing technical parameters for breast tissue assessment, providing guidelines for transducer positioning, standardized compression techniques, and specific imaging depth requirements (63). Quality assurance measures establish clear criteria for image acceptability and measurement reliability (64), including indicators for signal quality, compression levels, and image stability.

Equipment-related variations pose another barrier to standardization (65). Different manufacturers utilize various approaches to elastography, resulting in inconsistent measurement scales and reporting methods across systems. This technical heterogeneity complicates multi-center studies and meta-analyses, potentially hindering broader validation and acceptance of elastography as a standard diagnostic tool.

Standardized reporting systems have been implemented to ensure clear communication of elastographic findings across clinical settings (22, 23). These systems incorporate specific terminology and classification schemes that facilitate consistent documentation and interpretation, particularly beneficial for longitudinal monitoring and detecting progressive tissue changes.

Promising approaches to address standardization challenges include artificial intelligence and machine learning technologies, which may help compensate for operator variability and normalize measurements across diverse patient populations. The development of quality assurance metrics and real-time feedback systems represents a crucial direction for enhancing measurement standardization.

The future of breast elastography standardization will likely require a multifaceted approach that combines technological innovation with practical clinical considerations. Successfully addressing these challenges will be essential for the broader adoption of elastography as a standard tool in breast cancer diagnosis and monitoring (63). Ongoing refinement of these protocols will enhance elastography’s role in breast cancer diagnostics, improving its integration into multimodal imaging and bolstering early detection efforts.

### 5.2 Economic and accessibility considerations

The economic aspects of implementing and maintaining breast elastography systems present significant challenges that influence their adoption and integration into healthcare settings (66). These considerations include direct costs of equipment acquisition and maintenance, as well as broader implications for healthcare providers and patient care delivery.

A major barrier to elastography adoption is the initial capital investment, particularly for smaller healthcare facilities (67). The high cost of advanced ultrasound systems with elastography capabilities requires careful financial planning and return on investment analysis. This investment encompasses not only the equipment but also necessary accessories, software licenses, and facility modifications. Providers must evaluate patient volume, reimbursement potential, and expected clinical benefits to justify these substantial upfront costs. The decision becomes more complex given the rapid pace of technological advancement, which may lead to equipment obsolescence.

Ongoing operational costs add another layer of economic challenge in maintaining elastographic services (68). These expenses include regular maintenance, software updates, and calibration services necessary for optimal performance. In addition, facilities must account for costs related to staff training, quality assurance programs, and continuing education to keep pace with technological developments. The cumulative impact of these operational expenses significantly influences the long-term financial sustainability of elastography programs, especially in resource-limited settings.

Reimbursement challenges critically impact healthcare providers and patients (69). Insurance coverage for elastographic examinations varies across systems, creating uncertainty in revenue projections and barriers to patient access. Many systems struggle to obtain adequate reimbursement rates that reflect the true cost of providing elastographic services, including direct and indirect costs, such as additional examination time and specialized expertise. Developing appropriate billing codes and reimbursement structures is essential for ensuring the financial viability of elastography programs.

Workforce-related costs are another significant component of the economic equation. Specialized training and ongoing skill development require substantial investment in human resources. Facilities must consider the direct costs of training and the productivity impact during learning periods, as well as the potential need for additional staff to maintain patient throughput. The requirement for experienced personnel may necessitate higher compensation levels, further affecting the overall cost structure.

Quality assurance programs and documentation requirements also contribute to cost considerations. Facilities must allocate resources for comprehensive quality control systems, including performance monitoring and documentation protocols. These programs require dedicated personnel time and may necessitate additional software, adding to the overall cost burden. Balancing investment in quality assurance with financial constraints is crucial for maintaining accreditation.

Cost-effectiveness analysis plays a major role in justifying elastography implementation (70). Providers must demonstrate that additional costs are offset by improvements in diagnostic accuracy and better patient outcomes. Developing robust economic models that capture both direct cost savings and indirect benefits is essential for supporting investment decisions.

### 5.3 Education and training requirements

The successful implementation of breast elastography in clinical practice relies heavily on comprehensive education and training programs for healthcare professionals (71). These programs must address multiple levels of expertise, from basic technical skills to advanced interpretation, presenting unique challenges in medical education (72).

Training for breast elastography extends beyond traditional ultrasound education, focusing on the physical principles of tissue elasticity and mechanical properties. Healthcare providers need a solid understanding of elastographic imaging fundamentals, including strain patterns, shear wave propagation, and tissue mechanical behavior. This theoretical knowledge is essential for proper technique application and result interpretation, necessitating well-structured educational programs to bridge the gap between basic physics and clinical practice.

Clinical skills development is a challenging aspect of elastography training. Practitioners must master techniques for optimal image acquisition, such as proper transducer positioning, appropriate compression application, and accurate region-of-interest selection. These skills require extensive hands-on practice and supervised experience, making the learning curve steep, especially for those less experienced with conventional ultrasound techniques. Training programs must ensure adequate opportunities for practical experience while maintaining patient care quality.

Interpretation expertise is another critical component of elastography education. Providers must be proficient in recognizing normal tissue patterns, identifying artifacts, and distinguishing pathological findings on elastographic images. This requires exposure to a wide range of cases and correlation with other imaging modalities. Developing pattern recognition skills and diagnostic confidence typically necessitates significant time and experience, making structured training programs essential for progressive exposure to complex cases. In addition, practitioners must learn to integrate elastographic findings with conventional ultrasound for comprehensive diagnostic assessments.

Quality assurance training is vital in elastography education. Providers must understand technique standardization, regular calibration, and quality control measures. Training should cover proper documentation practices, measurement standardization, and reporting protocols. Ongoing education is necessary to maintain consistency across operators and facilities, especially as technology evolves.

Continuing education presents ongoing challenges in maintaining elastography expertise. The rapid evolution of technology necessitates regular updates to knowledge and skills. Providers must stay current with new developments and emerging clinical applications. Developing effective continuing education programs that accommodate busy clinical schedules while providing meaningful learning opportunities remains a significant challenge.

Multi-disciplinary training adds complexity to elastography education. Different healthcare providers, including radiologists, sonographers, and referring physicians, require varying levels of expertise. Training programs must be tailored to meet the specific needs of different roles while fostering effective communication and collaboration.

Looking ahead, factors such as the integration of artificial intelligence and virtual training platforms may influence elastography education requirements, necessitating ongoing adaptation and innovation in training approaches to ensure providers maintain the necessary expertise for effective utilization.

### 5.4 Artificial intelligence integration

Despite the recent advances discussed in Section 4.2, the development and validation of AI systems for elastography face significant challenges. The creation of robust AI models requires extensive, well-curated datasets that represent diverse patient populations, pathological conditions, and imaging conditions. These datasets must account for variations in tissue composition, patient demographics, and technical factors that influence elastographic measurements. The collection and standardization of such comprehensive datasets present logistical challenges, particularly considering patient privacy regulations and the need for accurate pathological correlation. Furthermore, ensuring that training data adequately represents rare conditions and edge cases remains a persistent challenge in developing clinically reliable AI systems.

The integration of AI systems into existing clinical workflows presents another layer of complexity (73). Healthcare providers must navigate the implementation of AI tools while maintaining efficient patient care processes. This integration requires careful consideration of human-machine interaction, with systems designed to augment rather than replace clinical expertise. The development of intuitive user interfaces and clear presentation of AI-derived insights becomes crucial for effective clinical adoption. Additionally, the real-time processing requirements of elastographic imaging pose technical challenges for AI implementation, necessitating powerful computing infrastructure while maintaining cost-effectiveness.

Limitations also exist in terms of data availability and quality. The reliance on extensive datasets means that any gaps, biases, or inconsistencies in the data can directly affect the performance and reliability of AI models (74). For example, if the training data lacks representation of certain demographic groups (such as age, ethnicity, or gender) or specific pathological conditions, the AI may perform poorly in real-world scenarios, leading to disparities in diagnostic accuracy. Studies have shown that AI algorithms trained on predominantly homogeneous datasets often exhibit reduced sensitivity and specificity when applied to more diverse populations, which can exacerbate health inequities (75). Moreover, the need for high-quality, annotated data increases the complexity of model training. Collecting and annotating such data can be resource-intensive and time-consuming, often requiring collaboration across multiple institutions and adherence to strict ethical guidelines. For example, obtaining large sets of labeled elastographic images necessitates expert input from radiologists or pathologists, which may not always be readily available. This process can delay the development of AI systems and impact their readiness for clinical use.

Clinical validation of AI-enhanced elastography systems represents a critical challenge that must be addressed through rigorous scientific study (76). The development of appropriate validation methodologies that can assess both the technical performance and clinical utility of AI systems is essential. Multi-center trials are necessary to evaluate system performance across different clinical settings and patient populations. These studies must demonstrate not only improved diagnostic accuracy but also practical benefits in terms of workflow efficiency and patient outcomes (77). The regulatory landscape for AI-enhanced medical devices adds another layer of complexity, requiring careful navigation of approval processes while ensuring patient safety and system reliability.

The explainability and transparency of AI decisions in elastographic analysis present ongoing challenges that affect clinical acceptance and trust (78). Healthcare providers need to understand the basis for AI-generated recommendations to maintain their role in clinical decision-making. The development of interpretable AI models that can provide clear reasoning for their conclusions becomes crucial for clinical implementation. This aspect becomes particularly important in cases where AI findings might contradict traditional clinical assessments, requiring clear mechanisms for resolution of such discrepancies.

Looking forward, the successful integration of AI in breast elastography will require continued collaboration between clinicians, researchers, and technology developers. The evolution of AI systems must balance the drive for improved diagnostic performance with practical clinical requirements and regulatory compliance. Future developments should focus on creating more adaptive AI systems that can learn from ongoing clinical use while maintaining reliability and consistency. The establishment of standardized evaluation frameworks and performance metrics will be crucial for comparing different AI solutions and ensuring their clinical validity. As these systems mature, their role in clinical practice will likely expand beyond simple diagnostic assistance to include more comprehensive functions such as risk assessment, treatment planning, and outcome prediction.

### 5.5 Special populations

The application of breast elastography in special patient populations and advanced clinical scenarios presents unique challenges and considerations that require careful attention to technical adaptation, interpretation modifications, and specific protocol development. These specialized applications demand enhanced understanding and tailored approaches to ensure optimal diagnostic outcomes.

Pediatric and adolescent populations represent a distinct challenge in breast elastography implementation (79, 80). The developing breast tissue in young patients exhibits different mechanical properties and anatomical characteristics compared to adult breast tissue, necessitating modifications in technique and interpretation criteria. The typically denser and more fibrous nature of young breast tissue requires careful adjustment of elastographic parameters and measurement techniques. Additionally, special considerations must be given to radiation exposure concerns, patient positioning challenges, and the psychological aspects of examining young patients. The development of age-specific protocols and reference values becomes crucial for accurate assessment in this population.

Pregnant and lactating patients present another unique subset requiring specialized approaches in elastographic examination (81, 82). The significant physiological changes in breast tissue during pregnancy and lactation alter tissue elasticity and mechanical properties, potentially affecting measurement accuracy and interpretation. The increased vascularity, edema, and structural modifications during these periods necessitate careful technique adaptation and consideration of potential artifacts. Healthcare providers must develop specific protocols that account for these physiological changes while maintaining diagnostic accuracy. The challenge extends to establishing appropriate reference ranges and interpretation criteria for this population, as normal tissue characteristics may differ significantly from non-pregnant states.

Elderly patients and those with significant comorbidities present distinct challenges in elastographic examination (83). Age-related changes in breast tissue composition and elasticity, combined with potential mobility limitations and positioning difficulties, require careful consideration in technique adaptation. The presence of multiple comorbidities may affect tissue properties and measurement reliability, necessitating modified approaches to image acquisition and interpretation. In addition, the potential impact of various medical conditions and treatments on tissue elasticity must be considered when evaluating elastographic findings.

Post-surgical and post-treatment breast tissue evaluation represents an advanced application requiring specialized expertise (32, 84). The altered tissue architecture, presence of surgical changes, and effects of radiation therapy can significantly impact elastographic measurements and interpretation. Healthcare providers must develop specific protocols for evaluating post-treatment changes, distinguishing between normal healing processes and potential recurrence. The challenge extends to establishing appropriate timing for elastographic examinations following various treatments and developing criteria for assessing treatment response.

Implant-adjacent tissue assessment presents unique technical and interpretative challenges in elastography. The presence of breast implants affects ultrasound wave propagation and tissue displacement patterns, requiring careful technique modification and specialized approaches to image acquisition. Healthcare providers must develop expertise in evaluating tissue adjacent to implants while accounting for potential artifacts and limitations in measurement accuracy. The development of specific protocols for implant-adjacent tissue assessment becomes essential for maintaining diagnostic reliability.

High-risk screening populations require careful consideration in elastographic application. Patients with genetic predisposition to breast cancer or strong family history may benefit from enhanced surveillance protocols incorporating elastography. However, the implementation of such screening programs requires careful consideration of examination frequency, technique standardization, and result interpretation in the context of risk assessment. The development of specific protocols for high-risk screening must balance the potential benefits of enhanced detection with resource utilization and cost considerations.

Looking toward future developments, several factors may influence the expansion of elastography applications in special populations. Technological advancements may provide new capabilities for addressing current limitations and expanding the range of clinical applications. The development of specialized transducers, automated analysis tools, and advanced processing algorithms may enhance the ability to evaluate challenging cases and special populations. Success in addressing these challenges will require ongoing research, protocol refinement, and validation studies to establish evidence-based approaches for various specialized applications.

## 6 Conclusion

Ultrasound elastography has established itself as a valuable tool in breast cancer detection and characterization, with continuing technological advances promising further improvements in clinical utility. The integration of artificial intelligence, development of 3D capabilities, and refinement of quantitative analysis methods represent significant steps forward in the evolution of this technology. The success of elastography in clinical practice depends on proper implementation, including standardized protocols, comprehensive training programs, and appropriate quality assurance measures. As technology continues to evolve, ongoing research will likely expand its applications and address current limitations, further enhancing its role in breast cancer care. The future of breast elastography appears promising, with potential applications extending beyond current use cases. Continued technological innovation, coupled with growing clinical evidence and experience, suggests an expanding role for elastography in comprehensive breast cancer care strategies.
